# Vacuoles, E1 enzyme, X-linked, autoinflammatory, somatic (VEXAS) syndrome presenting as recurrent aseptic peritonitis in a patient receiving peritoneal dialysis: a case report

**DOI:** 10.1186/s12882-024-03454-9

**Published:** 2024-01-11

**Authors:** Natsuki Fukuda, Daisuke Kanai, Kaoru Hoshino, Yuriko Fukuda, Ryutaro Morita, Yuki Ishikawa, Tomohiko Kanaoka, Yoshiyuki Toya, Yohei Kirino, Hiromichi Wakui, Kouichi Tamura

**Affiliations:** 1 Department of Nephrology, Yokohama Sakae Kyosai Hospital, Kanagawa, Japan; 2 Department of Medical Science and Cardiorenal Medicine, Yokohama City University Graduate School of Medicine, 3-9 Fukuura, Kanazawa-ku, Kanagawa, 236-0004 Japan; 3 Department of Nephrology, Yokohama Minami Kyosai Hospital, Kanagawa, Japan; 4 Department of Stem Cell and Immune Regulation, Yokohama City University Graduate School of Medicine, Kanagawa, Japan

**Keywords:** VEXAS syndrome, Peritoneal dialysis, Aseptic peritonitis, Giant cell arteritis, Relapsing peritonitis

## Abstract

**Background:**

Vacuoles, E1 enzyme, X-linked, autoinflammatory, somatic (VEXAS) syndrome is caused by mutations in the ubiquitin-activating enzyme1 (*UBA1*) gene and characterised by an overlap between autoinflammatory and haematologic disorders.

**Case presentation:**

We reported a case of a 67-year-Japanese man receiving peritoneal dialysis (PD) who had recurrent aseptic peritonitis caused by the VEXAS syndrome. He presented with unexplained fevers, headache, abdominal pain, conjunctival hyperaemia, ocular pain, auricular pain, arthralgia, and inflammatory skin lesions. Laboratory investigations showed high serum C-reactive protein concentration and increased cell count in PD effluent. He was treated with antibiotics for PD-related peritonitis, but this was unsuccessful. Fluorine-18-fluorodeoxyglucose (FDG) positron emission tomography/computed tomography images demonstrated intense FDG uptake in his left superficial temporal artery, nasal septum, and bilateral auricles. The working diagnosis was giant cell arteritis, and he was treated with oral prednisolone (PSL) 15 mg daily with good response. However, he was unable to taper the dose to less than 10 mg daily because his symptoms flared up. Since Tocilizumab was initiated, he could taper PSL dose to 2 mg daily. Sanger sequencing of his peripheral blood sample showed a mutation of the UBA1 gene (c.122 T > C; p.Met41Thr). We made a final diagnosis of VEXAS syndrome. He suffered from flare of VEXAS syndrome at PSL of 1 mg daily with his cloudy PD effluent. PSL dose of 11 mg daily relieved the symptom within a few days.

**Conclusions:**

It is crucial to recognise aseptic peritonitis as one of the symptoms of VEXAS syndrome and pay attention to the systemic findings in the patients.

**Supplementary Information:**

The online version contains supplementary material available at 10.1186/s12882-024-03454-9.

## Background

Vacuoles, E1 enzyme, X-linked, autoinflammatory, somatic (VEXAS) syndrome is a rare disorder which was newly described in December 2020. Myeloid lineage-restricted somatic mutations of the *UBA1* gene affecting the Met41 residue were detected in all patients with VEXAS syndrome [1]. It is an adult-onset systemic autoinflammatory syndrome with haematologic disorders and its symptoms are a combination of symptoms of rheumatologic, dermatologic, and haematologic diseases such as fevers, headache, conjunctival hyperaemia, ocular pain, auricular pain, Sweet’s syndrome, arthralgia, pleural effusion and cytopenia with morphological dysplasia of bone marrow and characteristic vacuoles of granulocytic and erythroid precursors [1].

We herein report our experience with a patient receiving peritoneal dialysis (PD) who had recurrent aseptic peritonitis caused by VEXAS syndrome.

## Case presentation

A 67-year-old man attended our department for evaluation of his turbid PD effluent. He also presented with sore throat, conjunctival hyperaemia, ocular pain, headache, and abdominal pain.

**Fig. 1:**
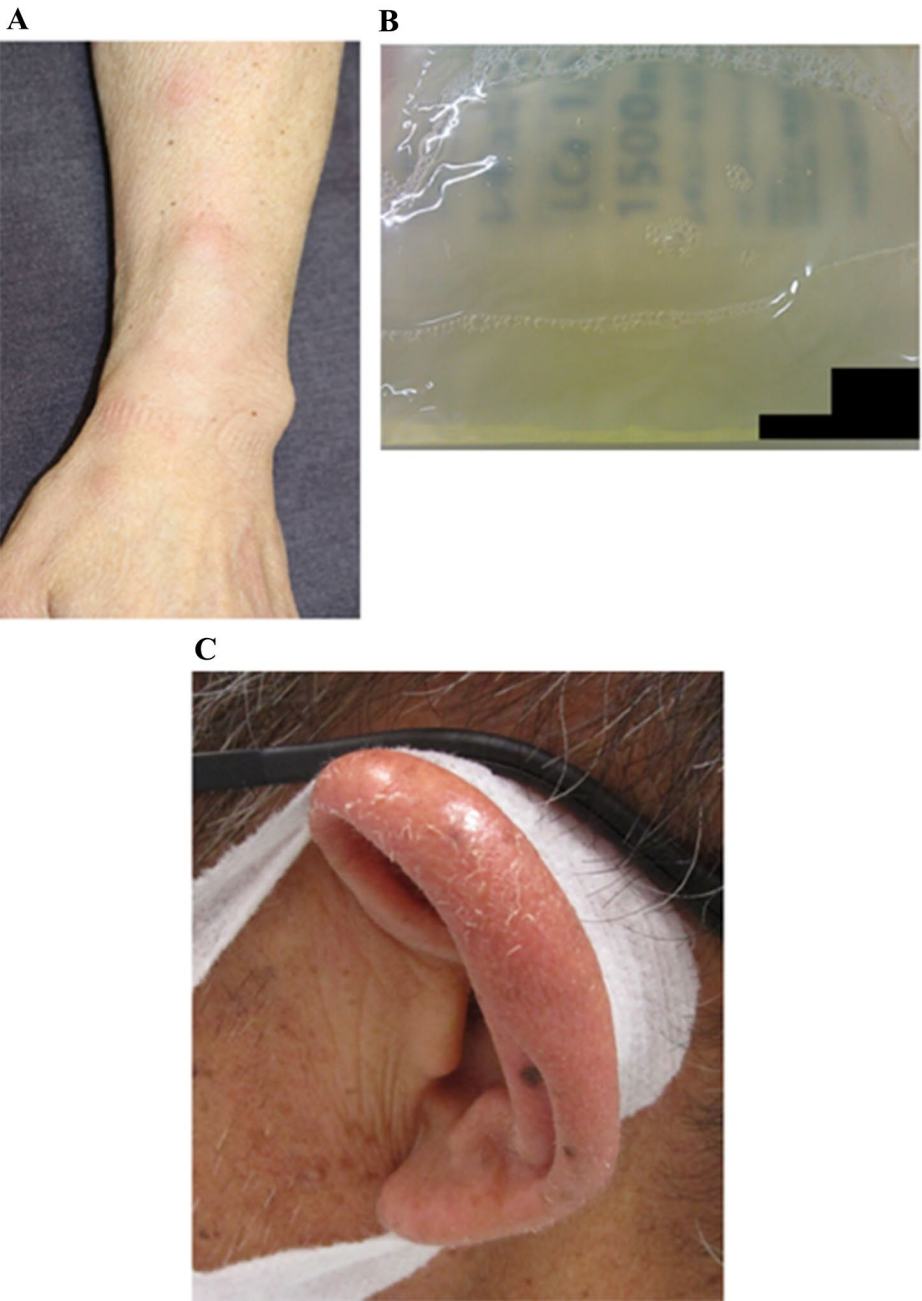
Clinical manifestations of VEXAS syndrome. (**A**) Erythema nodosum on the forearm. (**B**) Osteochondritis of the ear. (**C**) PD effluent turbidity. The letters on the back of the bag appear blurred due to the turbidity of the effluent. PD, peritoneal dialysis; VEXAS, Vacuoles, E1 enzyme, X-linked, autoinflammatory, somatic

Three months prior, he had noticed a sore throat, and upon examination by his primary care physician, he was diagnosed with upper respiratory tract infection and prescribed analgesics. The sore throat improved over the course of a few days. Two months before the current presentation, he also experienced redness and swelling on the left cheek, accompanied by heat sensation, and conjunctival hyperaemia in the right eye. Subsequently, he noted redness, swelling, pain, heat sensation extending from both eyelids to the cheek, and a fever of around 37.5 °C. He sought consultation with the dermatology department, where the serum CRP level was 7.36 mg/dL, and a diagnosis of cellulitis was made. He was initiated on oral antibiotics (amoxicillin hydrate). One week later, the skin lesions had diminished, but a tender erythematous plaque measuring 5 cm in diameter was newly observed in his right cheek region. With continued oral amoxicillin for another week, the skin lesions resolved. However, painful erythematous plaques with infiltrative features, measuring approximately 2 cm in diameter, newly appeared on the forehead and both auricles, accompanied by an increase in CRP levels to 9.97 mg/dL. He was admitted to dermatology department of our hospital to evaluate inflammatory skin lesions (Fig. 1a).

A skin biopsy was performed, and the histopathological examination confirmed the diagnosis of nodular erythema. Treatment with oral prednisolone (PSL) at a dosage of 20 mg per day was initiated, resulting in a prompt resolution of the skin lesions. The PSL was gradually tapered over a period of 2 months and eventually discontinued. However, thereafter, the CRP levels began to rise again, and he experienced turbidity in the PD effluent, along with headaches and conjunctival hyperaemia.

His medical history of kidney disease was as follows: since childhood, he had been experiencing haematuria. At the age of 20, a family history of polycystic kidney disease (PKD) was identified through his father, who had multiple cystic kidneys. Subsequent imaging studies led to the diagnosis of autosomal dominant polycystic kidney disease (ADPKD). At the age of 49, the patient underwent clipping surgery for a cerebral aneurysm. At the age of 60, he suffered from a right subcortical haemorrhage, accompanied by creatinine levels of 1.99 mg/dL, an estimated glomerular filtration rate (eGFR) of 28.2, and a urine protein-to-creatinine ratio (UPCR) of 0.5 g/gCr. At the age of 61, tolvaptan was introduced, considering a total kidney volume of 2759 mL, a kidney volume growth rate of 22%. At the age of 64, creatinine levels rose to 4.26 mg/dL, eGFR dropped to 12.0, and UPCR increased to 1.25. The patient commenced peritoneal dialysis at the age of 65. He also suffered from polymyalgia rheumatica at age 57.

When he attended our department, he presented with PD effluent turbidity (Fig. 1b). He also had no skin lesion, no arthralgia, no findings of PD catheter exit-site infection or tunnel infection. His vital signs were as follows: body weight, 63.5kg; body temperature, 37.0 °C; blood pressure (BP), 101/61 mmHg; heart rate (HR), 70 beats/min; respiratory rate, 30 breaths/min. On physical examination, some lymph nodes were swollen—including bilateral cervical, supraclavicular fossa, subclavian, axillary, and inguinal lymph nodes about 1–2 cm in size with mobility, but without calor and tenderness. Laboratory investigations showed the following: C-reactive protein (CRP) concentration 13.43 mg/dL (normal < 0.3 mg/dl); white blood cell count of the PD effluent, 275/µL with 80% polymorphonuclear cells. He was clinically diagnosed with PD-related bacterial peritonitis. We administered Cefepime (CFPM) of 0.5 g every 24 h for five days but observed no improvement in his CRP levels and PD effluent cell count. Subsequently, we added Vancomycin (VCM) of 1 g every 24 h. However, despite the treatment, his clinical symptoms, CRP levels, and PD effluent cell counts did not improve. Considering the ineffectiveness of the antibiotic therapy, both CFPM and VCM were discontinued on the 10th day of admission. On the same day, scattered nodular erythema-like rashes were observed on both forearms. From the 11th day of admission, we administered Ceftazidime (CAZ) of 1 g every 24 h for 5 days, but neither symptoms nor test results showed improvement. Furthermore, the severity of headaches and conjunctival hyperaemia worsened, and nodular erythema-like rashes appeared on the head and trunk. Suspecting antibiotic-resistant bacterial peritonitis, we conducted repeated bacterial culture tests on PD effluent and blood, all of which returned negative results. Consultation with a rheumatologist raised suspicion of involvement of an autoimmune inflammatory disorder. From the 16th day, we initiated treatment with oral colchicine 0.5 mg/day. Colchicine improved symptoms such as headaches, conjunctival hyperaemia, and skin rash, and showed a decrease in CRP levels and PD effluent cell count. However, the CRP level did not drop below 9 mg/dL, and the PD effluent cell count did not decrease to less than 75 cells/µL. Therefore, from the 20th day, the colchicine dose was increased to 1 mg/day, but the improvement in symptoms and data was limited, and the appearance of nausea due to colchicine was noted.

**Fig. 2:**
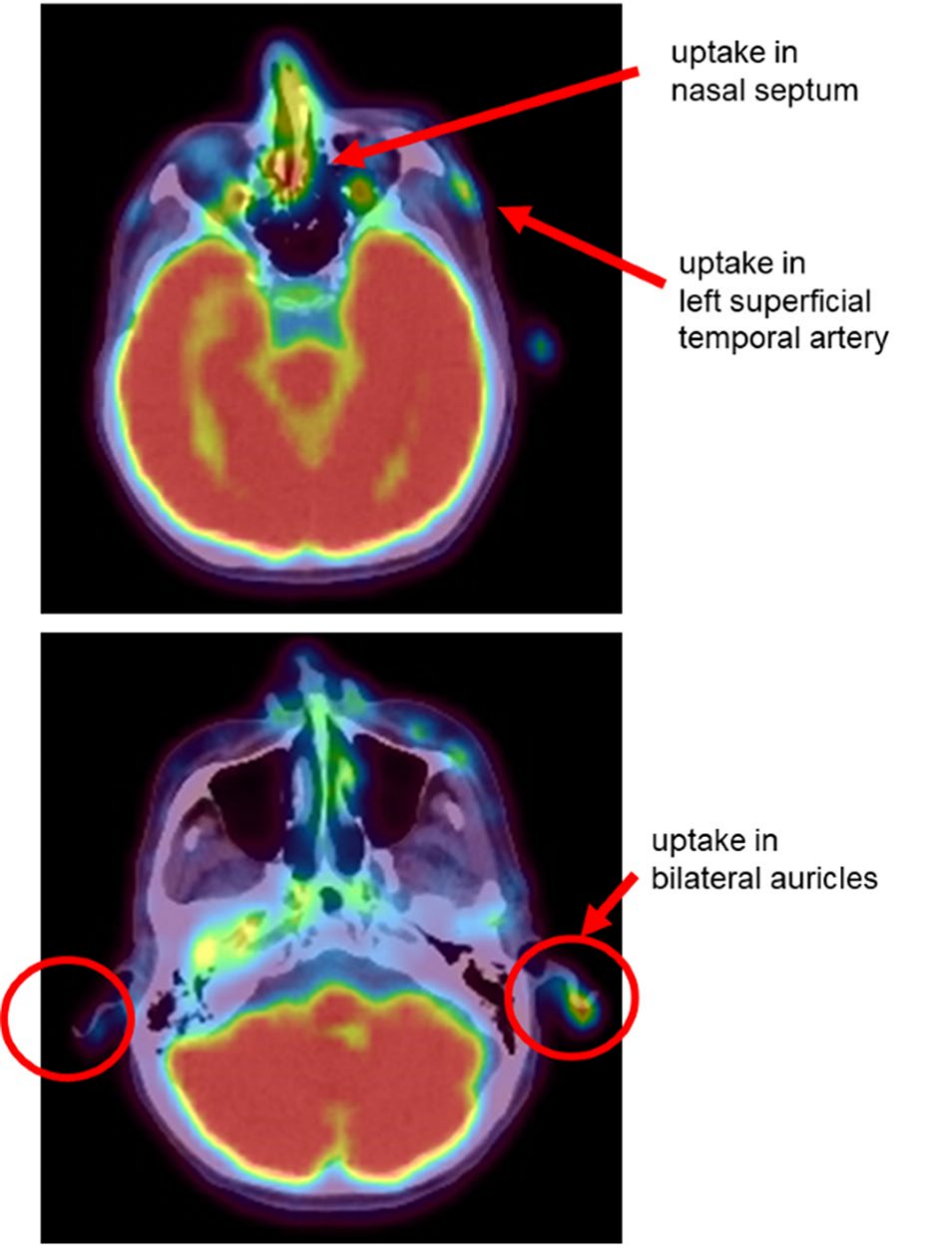
^18^F-FDG PET/CT images of the patient’s head. ^18^F-FDG PET/CT images demonstrated intense FDG uptake in his left superficial temporal artery, nasal septum, and bilateral auricles

We performed Fluorine-18 Fluorodeoxyglucose-Positron-Emission Tomography/Computed Tomography (^18^F-FDG PET/CT) to evaluate the systemic inflammation. ^18^F-FDG PET/CT images demonstrated intense symmetric FDG uptake in his left superficial temporal artery, nasal septum, and bilateral auricles (Fig. 2). Mild uptake was observed in abdominal lymph nodes, left axillary lymph node, and cervical lymph node. Three of five American College of Rheumatology (ACR) classification criteria for giant cell arteritis (GCA) including age at onset > 50 years, new-onset headache, and erythrocyte sedimentation rate > 50 mm/hour were met and he was classified as GCA.

Furthermore, Four of six McAdams criteria classification criteria for relapsing polychondritis (RP) including intense symmetric FDG uptake in his nasal septum and bilateral auricles on ^18^F-FDG PET/CT, inflammation of the eye, and bilateral sensorineural hearing loss. He also classified as RP.

From the 26th day, colchicine was reduced to 0.5 mg per day, and prednisolone (PSL) at a dosage of 15 mg per day was added. Following the initiation of PSL therapy, clinical symptoms of GCA and RP disappeared, the patient’s hearing showed improvement on the audiogram, with a decrease from 46.3 dB to 37.5 dB in the right ear and from 51.3 dB to 35 dB in the left ear, and both CRP levels and PD effluent cell counts returned to within normal range. Considering the coexistence of multiple autoimmune diseases such as GCA, RP, and aseptic serositis (peritonitis), VEXAS syndrome was suspected.

Sanger sequencing of his peripheral blood sample showed a mutation affecting methionine-41of the *UBA1* gene (c.122 T > C; p.Met41 threonine [Thr]), and we made a final diagnosis of VEXAS syndrome.

At the PSL dose of 9 mg daily, he suffered from loss of appetite and his serum CRP concentration was increased. It took about 3 months to reduce the dose from 15 mg to 9 mg daily. Tocilizumab (TCZ) 500 mg(8 mg/kg)/month was initiated as a steroid-sparing agent and shortly enabled him to achieve remission again.

After 5 weeks of treatment with TCZ, he developed conjunctival hyperaemia, arthralgia, auricular chondritis (Fig. 1c), and inflammatory skin lesions such as Sweet’s syndrome on his upper limbs and neck at the PSL dose of 1 mg daily and turbidity of his PD effluent was also observed. Laboratory investigation showed an elevated serum CRP concentration 1.72 mg/dL, dialysate cell count, 550/µL but culture of his PD effluent was negative.

**Fig. 3:**
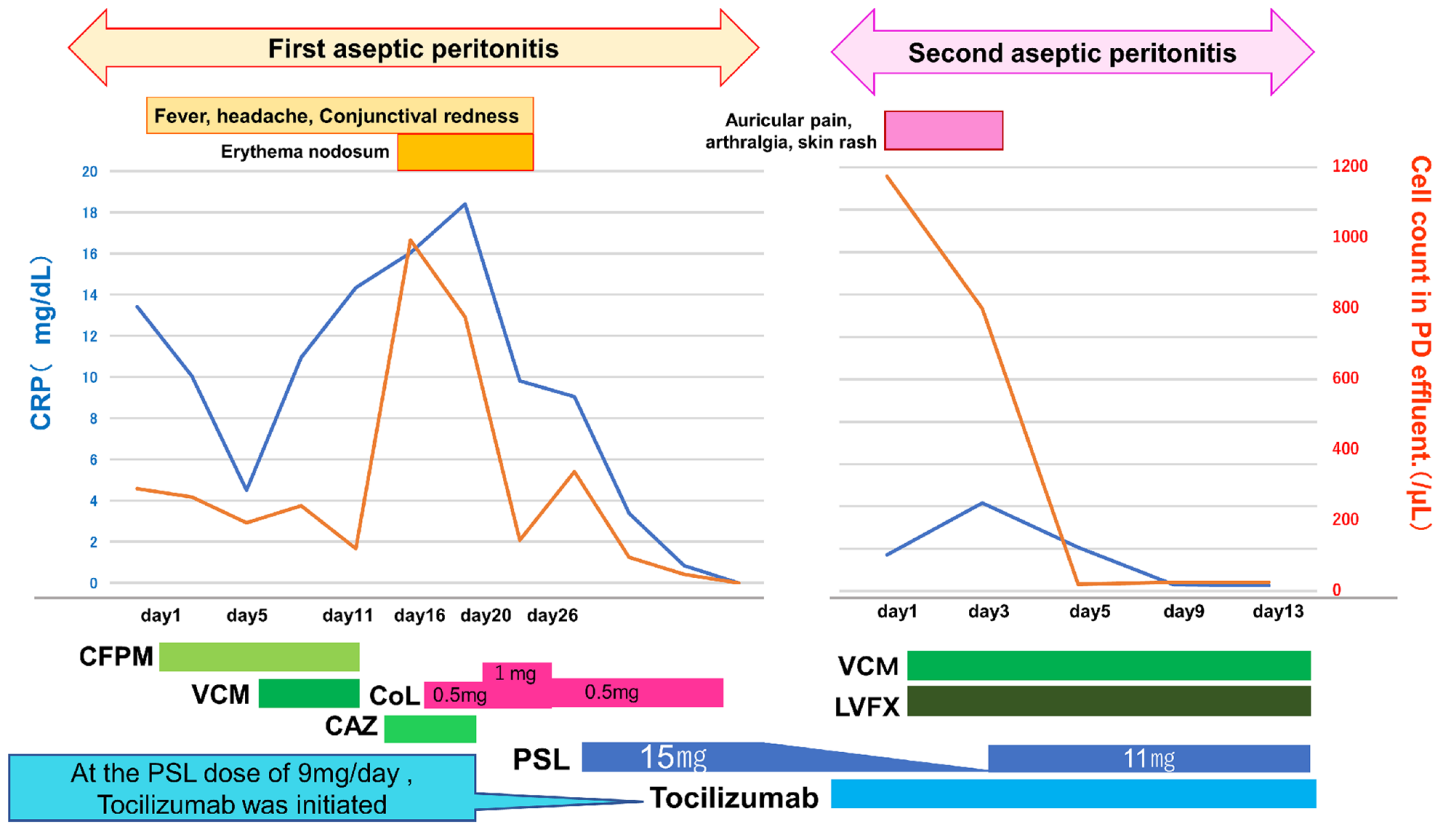
Clinical course diagram. Abbreviations: CAZ, ceftazidime; CFPM, cefepime; COL, colchicine; CRP, C-reactive protein; LVFX, levofloxacin; PD, peritoneal dialysis; PSL, prednisolone; TCZ, Tocilizumab; VCM, vancomycin

Considering the appearance of conjunctival hyperaemia, skin lesions resembling Sweet’s syndrome, and symptoms of RP, a recurrence due to VEXAS syndrome was contemplated. However, because the patient was on PD, and PD-related bacterial peritonitis could not be ruled out, initial treatment involved administering levofloxacin (LVFX) at 500 mg on the first day, followed by 250 mg every other day, in conjunction with VCM at 1 g every 24 h. Nevertheless, there was no improvement in clinical symptoms, skin lesions, or PD effluent turbidity. On the third day, PSL was increased to 11 mg per day. Subsequently, the PD effluent cell count improved and returned to within normal range by the fifth day, with resolution of conjunctival hyperaemia, joint pain, chondritis of the auricle, and inflammatory skin lesions appearing on the upper limbs and neck by the sixth day. Furthermore, the serum CRP level normalised by the ninth day (Fig. 3).

## Discussion and conclusions

This was a rare case of a patient receiving PD who had experienced recurrent aseptic peritonitis due to VEXAS syndrome. Peritonitis was easily detected since the patient directly visualised cloudy PD effluent. To the best of our knowledge, there was no report of patients on PD with VEXAS syndrome and no report of patients with aseptic peritonitis caused by VEXAS syndrome (Supplementary Table). The patient also had relapsing polychondritis, which caused his auricular lesions and scleritis; giant cell arteritis, which caused headache; Sweet’s disease, which caused inflammatory skin lesions. A mutation at methionine-41of the *UBA1* gene (c.122 T > C; p.Met41Thr) was observed in the patient’s peripheral blood sample.

VEXAS syndrome was first described in December 2020 [1]. It is characterised by a somatic mutation in p.Met41 of the gene ubiquitin-activating enzyme1 (*UBA1*) in an X chromosome [1] and is an autoinflammatory syndrome with acquired onset in late adulthood [2].

Ubiquitination is a proteolytic system catalysis involving three enzymes: ubiquitin activating enzyme (E1 enzyme), ubiquitin binding enzyme (E2 enzyme), and ubiquitin ligase (E3) [3]. Ubiquitination involves variations of ubiquitin chains and branches and is known to play a role in the degradation of unwanted proteins to control signal transduction during cell survival, immunity, and inflammation [4, 5]. In VEXAS syndrome, a somatic mutation in p.Met41 of the *UBA1* gene causes messenger RNA (mRNA) transcription to start at p.Met67, which prevents normal ubiquitination signalling and leads to systemic inflammation [1]. There are three patterns of somatic mutations: leucine mutation, valine mutation, and threonine mutation. Threonine is the most frequent substitutional amino acid caused by the somatic mutation [1]. According to a French case analysis, 5-year survival rates in patients depend on their mutation: the leucine mutation has the highest rate, 100%; valine mutation, 76.7%; and threonine mutation, 83.1%. The leucine mutation is associated with mild to moderate phenotype, possibly resulting in a better overall prognosis [6]. Although phenotypic differences in gene mutations have not yet been clearly reported, patients with RP, especially those with skin lesions, are more likely to have *UBA1* gene mutations, VEXAS syndrome [7]. The patient also had skin lesions and was noted to have RP.

**Fig. 4:**
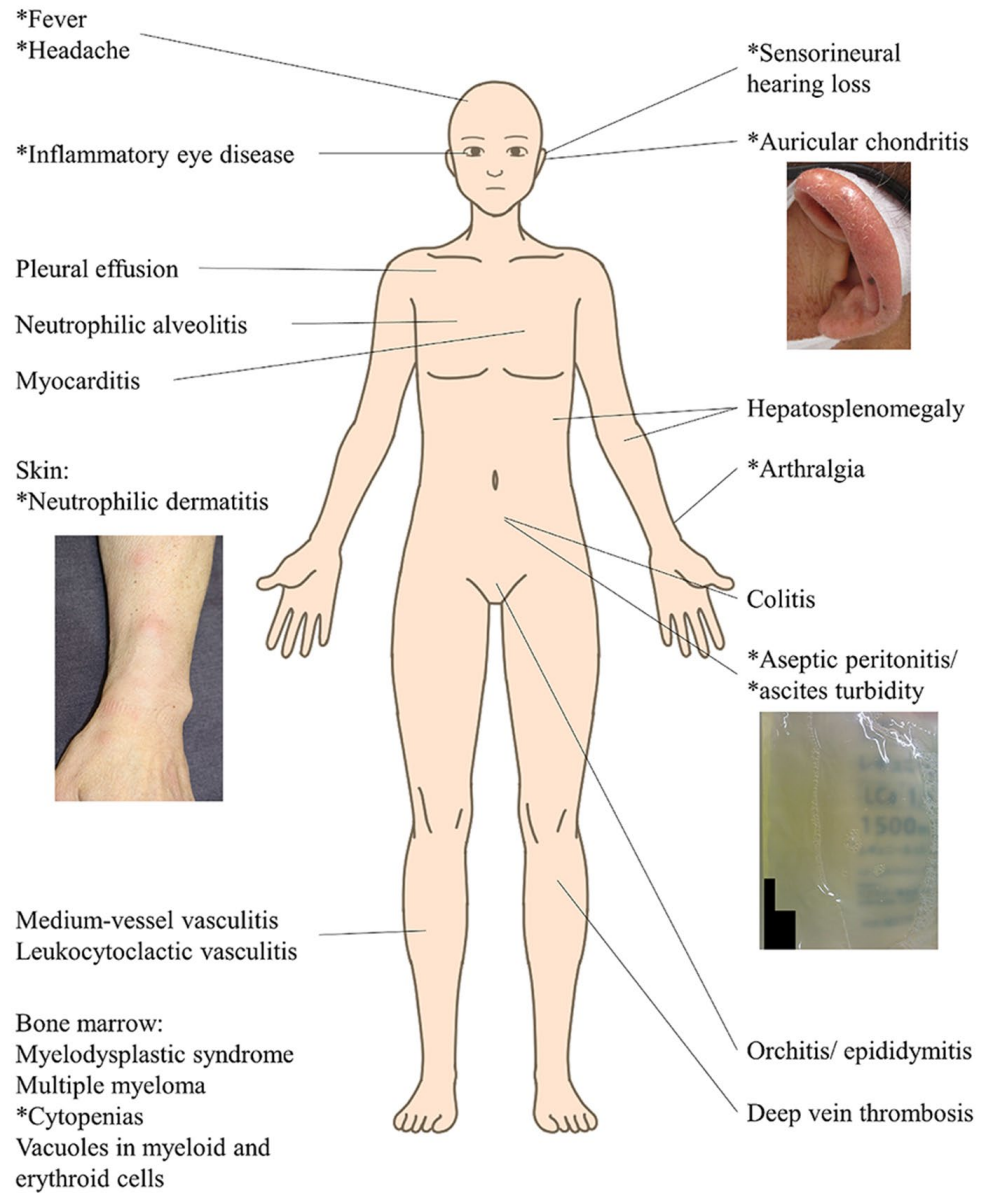
A wide range of inflammatory symptoms in VEXAS syndrome. The asterisk (*) in the figure indicates the symptoms present in our patient. VEXAS, Vacuoles, E1 enzyme, X-linked, autoinflammatory, somatic

The symptoms of VEXAS syndrome are a combination of symptoms of variable diseases including rheumatologic, dermatologic, and haematologic diseases: relapsing polychondritis, 46%; polyarteritis nodosa, 9%; Sweet’s disease, 46%; myelodysplastic syndromes (MDS), 37%; multiple myeloma, 12.5% [1,2, 8−10] (Fig. 4). Although kidney injury in VEXAS syndrome were mentioned in some cases, most of them seemed to be secondary to other co-morbidities (Supplementary Table). Only one case of interstitial nephritis due to VEXAS syndrome was reported thus far [11]. GCA was also a rare complication in VEXAS syndrome [12]. Vacuolated myeloid precursors in bone marrow aspirate or biopsy are often observed [1,2]; however, the patient had not received bone marrow investigation.

Patients with VEXAS syndrome are diagnosed with rheumatic diseases such as RP, Sweet’s syndrome, polyarteritis nodosa, GCA, or blood disorders such as myelodysplastic syndrome (MDS) and multiple myeloma (MM). Phenotypes vary, but the presence of a genetic variant of *UBA1* in a genetic diagnosis is a definitive diagnosis [13].

The patient was classified as having recurrent polychondritis following McAdam’s criteria, which included intense symmetric FDG uptake in his nasal septum and bilateral auricles on ^18^F-FDG PET/CT [14], inflammation of the eye, and bilateral sensorineural hearing loss. The patient’s classification as RP led to testing for genetic mutations in peripheral blood cells, which led to the diagnosis of VEXAS syndrome.

Treatment of VEXAS syndrome involves the suppression of the initial inflammation with steroids, followed by Disease-Modifying Antirheumatic Drugs (DMARDs) and immunosuppressive drugs to reduce the steroid dose [15−17]. Bone marrow transplantation is also considered as a radical treatment [18]. In a French case report, steroids were used in 74% of cases, conventional DMARDs in 18.2%, and biological agents in 33.1%. Of these, 45.7% of the cases led to steroid dependence, and the average dose used was 20 mg daily [6]. In this case, the patient could not reduce the PSL dose lower than 15 mg daily and TCZ was started to decrease the dose of PSL. TCZ approved for the treatment of inflammatory diseases, such as rheumatoid arthritis and GCA, is reported to be useful in managing severe inflammation in VEXAS syndrome and preserving the cumulative dose of PSL [16].

When peritonitis is observed in patients on PD, it is important to consider the possibility of aseptic peritonitis due to autoimmune diseases, including VEXAS syndrome, and pay attention to the systemic findings.

### Electronic supplementary material

Below is the link to the electronic supplementary material.

**Supplementary Material 1:** Review of literature of VEXAS syndrome

## Data Availability

The data and materials are available from the corresponding author, upon reasonable request.

## Abbreviations

ACR: American College of Rheumatology
ADPKD: autosomal dominant polycystic kidney disease
BP: blood pressure
CAZ: ceftazidime
CFPM: cefepime
CRP: C-reactive protein
DMARDs: Disease-Modifying Antirheumatic Drugs
^18^F-FDG PET/CT: Fluorine-18-Fluorodeoxyglucose Positron-Emission Tomography/Computed Tomography
GCA: giant cell arteritis
HR: heart rate
LVFX: levofloxacin
MDS: myelodysplastic syndrome
Met: methionine
MM: multiple myeloma
mRNA: messenger RNA
PD: peritoneal dialysis
PSL: prednisolone
PKD: polycystic kidney disease
RP: relapsing polychondritis
TCZ: Tocilizumab
Thr: threonine
UBA1: ubiquitin-activating enzyme1
UPCR: urine protein-to-creatinine ratio
VCM: vancomycin
VEXAS: Vacuoles, E1 enzyme, X-linked, autoinflammatory, somatic
