# Supplementary material for: Vacuoles, E1 enzyme, X-linked, autoinflammatory, somatic (VEXAS) syndrome presenting as recurrent aseptic peritonitis in a patient receiving peritoneal dialysis: a case report
Source: BMC Nephrol. 2024 Jan 11;25:18. doi: 10.1186/s12882-024-03454-9 (PMC10785490; doi:10.1186/s12882-024-03454-9)
Supplement: Supplementary file 1 — Supplementary Material 1: Review of literature of VEXAS syndrome [file 12882_2024_3454_MOESM1_ESM.docx]

**Supplementary Table Review of literature of VEXAS syndrome**

The data presented in the Supplementary Table were curated through a PubMed search and retrieval of references from pertinent articles utilizing the search term ‘VEXAS’. This table exclusively incorporates articles featuring and detailing newly reported cases of VEXAS syndrome. Inclusion criteria were limited to articles published in English from the inception of records to August 31, 2022, and restricted to human studies. While a few cases of pleural effusion or pericardial effusion were identified, there were no documented reports of ascites or peritonitis attributed to VEXAS syndrome.
